# Epigenetic and genetic variation among three separate introductions of the house sparrow (*Passer domesticus*) into Australia

**DOI:** 10.1098/rsos.172185

**Published:** 2018-04-11

**Authors:** E. L. Sheldon, A. Schrey, S. C. Andrew, A. Ragsdale, S. C. Griffith

**Affiliations:** 1 Department of Biological Sciences, Macquarie University, Sydney, New South Wales 2109, Australia; 2 Department of Biology, Armstrong State University, Savannah, GA, USA

**Keywords:** DNA methylation, invasive species, house sparrow, epigenetic variation, epigenetics, introduction history

## Abstract

Invasive populations are often associated with low levels of genetic diversity owing to population bottlenecks at the initial stages of invasion. Despite this, the ability of invasive species to adapt rapidly in response to novel environments is well documented. Epigenetic mechanisms have recently been proposed to facilitate the success of invasive species by compensating for reduced levels of genetic variation. Here, we use methylation sensitive-amplification fragment length polymorphism and microsatellite analyses to compare levels of epigenetic and genetic diversity and differentiation across 15 sites in the introduced Australian house sparrow population. We find patterns of epigenetic and genetic differentiation that are consistent with historical descriptions of three distinct, introductions events. However unlike genetic differentiation, epigenetic differentiation was higher among sample sites than among invasion clusters, suggesting that patterns of epigenetic variation are more strongly influenced by local environmental stimuli or sequential founder events than the initial diversity in the introduction population. Interestingly, we fail to detect correlations between pairwise site comparisons of epigenetic and genetic differentiation, suggesting that some of the observed epigenetic variation has arisen independently of genetic variation. We also fail to detect the potentially compensatory relationship between epigenetic and genetic diversity that has been detected in a more recent house sparrow invasion in Africa. We discuss the potential for this relationship to be obscured by recovered genetic diversity in more established populations, and highlight the importance of incorporating introduction history into population-wide epigenetic analyses.

## 1. Introduction

Invasive species offer an opportunity to investigate rapid evolution in novel environments; however, they also challenge our understanding of the process of adaptation. Invasive populations are often associated with low levels of genetic diversity, owing to population bottlenecks at the initial stages of invasion [1]. Reduced genetic variation is expected to constrain the evolutionary potential of a given population [2,3]; yet, many introduced populations are successful, and well able to adapt to non-native environments. The expansion of invasive species across novel environments thus presents an ‘*invasive paradox*’ [4,5].

Epigenetic mechanisms (phenomena that alter gene expression without changing DNA sequences) can contribute to phenotypic variation [6–8], and have recently been proposed to facilitate the success of invasive species in novel environments, by compensating for reduced levels of additive genetic variation [9,10]. The most widely studied epigenetic mechanism is DNA methylation, which refers to the addition of a methyl group to a cytosine base, most often when the cytosine is immediately followed by a guanine on the DNA sequence (i.e. CpG sites; [11]). Because CpG sites are enriched in regulatory sequences, variation in DNA methylation can alter gene expression, and potentially affect ecologically relevant phenotypes, without changing the underlying DNA sequence [12–15].

Genome-wide patterns of DNA methylation have substantially higher mutation rates than DNA sequences [16], and can be induced stochastically (via re-patterning errors during DNA replication; [17]) and in response to local environmental stimuli during an organism's lifetime [18–21]. Owing to their links with phenotypic variation, environmentally induced epigenetic variation and stochastic epimutations have been proposed to be among the potential mechanisms underlying phenotypic plasticity and diversifying bet hedging strategies, respectively [22]. Such flexibility in phenotypic expression, in the absence of underlying genetic variation, could be particularly beneficial to invasive populations exposed to novel environments [16,23].

Relatively little is known about the role of epigenetic variation in mediating adaptation and plasticity in invasive, or introduced vertebrate species. However, the few studies available indicate that DNA methylation may play an important role in the ability of genetically depauperate populations to adapt to novel environments [1]. For example, local environmental conditions have been shown to elicit persistent epigenetic changes in an invasive, clonal fish species (*Chrosomus eos-neogaens*), resulting in a great deal of epigenetic differentiation among different habitats [24]. Pairwise comparisons of epigenetic and genetic differentiation between different habitats in an invasive plant species have also indicated that epigenetic marks can differentiate in response to local conditions, potentially contributing to phenotypic diversity independently of genetic differentiation [9]. Levels of epigenetic diversity have also been suggested to contribute to invasive expansion, by acting as an alternative source of variation in areas with low genetic diversity. One of the first studies of a terrestrial vertebrate to examine this idea was the study of the recent house sparrow (*Passer domestics*), introduction into Kenya, Africa, by Liebl *et al*. [10]. In this study of seven populations, from a single founding event around half a century ago, a negative relationship between epigenetic and genetic diversity was detected, suggesting that epigenetic diversity may act as a compensatory source of phenotypic variation when genetic diversity is reduced in the initial stages of invasion [10].

Preceding the later introduction into Kenya by *ca.* 100 years, the house sparrow was also deliberately introduced into Australia in the 1860s, via numerous shippings from Europe and India by the ‘Australian Acclamation Society’ [25]. The invasion history of the Australian house sparrow population has been previously described using historical information [25], and molecular evidence [26]. These studies identified three separate introduction events into Australia (into: Melbourne (VIC), Adelaide (SA), and Brisbane (QLD)) and two isolated translocations from Melbourne to Sydney (NSW) and Hobart (Tasmania), all occurring in the 1860s. These multiple introduction events provide an opportunity to examine the extent to which epigenetic patterns observed in an earlier study of the same species [10] are replicated across multiple and distinct introduction events.

Using epigenetic data from methylation-sensitive-amplification fragment length polymorphism (MS-AFLP), we compare epigenetic differentiation between Australian house sparrows (*Passer domesticus*) with genetic differentiation using microsatellite data from a previous description of the Australian house sparrows genetic population structure [26]. We first examine whether patterns of epigenetic variation across 15 different sites throughout Australia align within three population clusters that can be linked to the three historically independent introduction events [25,26]. We predict that if epigenetic marks are affected by different founding events, then epigenetic variation will be structured by the three introductions. By expanding on the study by Liebl *et al.* [10], we then test whether the negative, potentially compensatory relationship between genetic and epigenetic diversity observed in the introduced African population exists across multiple introduction events in the Australian population of house sparrows.

## 2. Material and methods

### 2.1. Sampling

**Figure 1.**
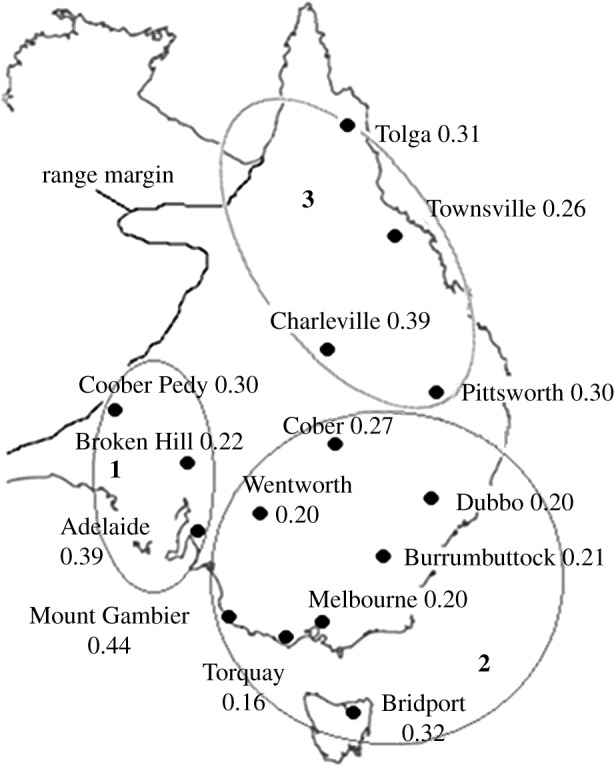
Map of the Eastern half of Australia labelled with the 15 study sites and their corresponding epigenetic diversity (epi-h) values. Sites derived from the same introduction event are grouped within an oval; 1, the South Australia introduction; 2, the Victoria/New South Wales introduction; 3, the Queensland introduction. The house sparrows estimated range edge is also plotted.

As part of a broader study, blood samples were collected from adult house sparrows in multiple populations across the entire range of the house sparrow population in Australia. For this study, we initially selected 16 populations to screen for epigenetic variation (table 1 for sample sizes); Tolga, Townsville, Charleville, Pittsworth, Dubbo, Cobar, Wentworth, Burrumbuttock, Melbourne, Torquay, Bridport, Mt Gambier, Broken Hill, Adelaide and Cooper Pedy (figure 1). A blood sample (less than 50 µl) was taken from each individual at the time of capture and preserved thereafter in 95% ethanol at room temperature. DNA was extracted using the Gentra Puregene tissue kit (Qiagen, Valencia, CA, USA) and was stored in 40 µl of TE buffer. We aimed to screen variation in DNA methylation for the 24 samples with the most optimal DNA yields from each population selected (above), and attempted to amplify and score microsatellites for around 40 individuals in each population with the exception of two (Coober Pedy and Burrumbuttock in which only 20 and 25 samples were available, respectively).

**Table 1.** Introduction clusters and sites where house sparrows were collected and screened for variation in DNA methylation. (The number of individuals screened for variation in DNA methylation/genetic variation is represented by *N*_epi_/*N*_G_. Epigenetic diversity is shown as haplotype diversity (epi-h) and percentage of polymorphic loci (%Poly), and genetic diversity is shown as expected heterozygosity (He), the mean number of alleles detected in an introduction cluster (Na)-indicated by an asterisk, and mean allelic richness within a site (Ar).)

introduction clusters and sites	*N*_epi_/N_G_	epi-h	%Poly	He	Ar/Na*
*Queensland cluster*	*29/103*	*0.36*	*82.11*	*0.814*	*15*.00*
Tolga	23/42	0.31	78.04	0.73	9.39
Townsville	16/42	0.26	90.24	0.73	9.78
Charleville	11/43	0.39	97.56	0.72	9.55
Pittsworth	11/42	0.30	90.24	0.77	10.83
*NSW and VIC cluster*	*90/307*	*0.29*	*69.51*	*0.834*	*19*.91*
Dubbo	9/39	0.20	60.98	0.79	10.63
Cobar	9/38	0.27	73.17	0.82	12.11
Wentworth	10/39	0.20	53.66	0.83	13.62
Burrumbuttock	16/25	0.21	68.29	0.83	11.20
Melbourne	17/42	0.20	75.61	0.82	12.94
Torquay	12/40	0.16	46.34	0.85	14.27
Bridport	9/43	0.32	80.49	0.82	11.04
Mount Gambier	8/41	0.44	97.56	0.79	11.49
*South Australian cluster*	*61/169*	*0.37*	*89.02*	*0.750*	*14*.46*
Broken Hill	9/41	0.22	70.73	0.81	11.84
Adelaide	8/42	0.39	90.24	0.81	10.97
Coober Pedy	12/20	0.30	85.36	0.77	7.69

### 2.2. Microsatellite genotyping

Samples were genotyped using two multiplexes developed by Dawson *et al*. [27], which included 11 polymorphic loci and a sexing locus (Multiplex 1: Ase18, Pdoµ1, Pdoµ3, Pdoµ6, Pdo10, P2D/P8; Multiplex 2: Pdo16A, Pdo17, Pdo19, Pdo22, Pdo27, Pdo40A). PCRs were carried out using 5 µl reactions. For each reaction, 1 µl of genomic DNA (approx. 100 ng µl^−1^) was added to 2.5 µl of Master Mix (Qiagen), 0.5 µl of primer mix (see concentrations in [27]) and 1 µl of Milli-Q water. Both multiplex reactions used the same PCR thermal cycle with a hot-start denaturing phase of 10 min at 95°C followed by 33 cycles of 94°C for 30 s, 57°C for 90 s and 72°C for 90 s, before a final extension at 72°C for 10 min. The post-PCR product was diluted and genotyped on the ABI 3730XL using GS500 (LIZ) as a size standard for Multiplex 1 and GS1200 for Multiplex 2. Microsatellite alleles were scored using the GeneMapper program v. 3.7. See full dataset in [26] which describes the genetic population structure in Australia and New Zealand.

### 2.3. DNA methylation using methylation sensitive-amplification fragment length polymorphism

We screened samples for variation in DNA methylation using MS-AFLP, which modifies the standard AFLP protocol by substituting the MseI enzyme with the methylation-sensitive isoschizomeric enzymes MspI and HpaII (New England Biolabs). Enzymes MspI and HpaII vary in their sensitivity to cytosine methylation. Cleaving by MspI is blocked when the inner cytosine is methylated, whereas cleaving by HpaII is blocked when either both cytosines are fully or hemi-methylated [28]. Together four types of variation can be scored; type 1 is when both enzymes cut at the restriction site and indicates no methylation; type 2 is when MspI does cut and HpaII does not cut, indicating the restriction site has a methylated internal cytosine C; type 3 is when MspI does not cut and HpaII does cut indicating the restriction site has a methylated outer C; and type 4 is when neither enzyme cuts indicating either both cytosines are methylated or the restriction site has mutated [9]. We treated type 4 as missing because the underlying methylation state cannot be determined. Recently, there has been a suggestion that types 2 and 3 should be analysed as separate states [29]; however, the actual source of these types of variation may be more complicated based on nested fragments [30]. As such, we combined types 2 and 3 into one methylated category, and treated all other states as not methylated. Throughout, we refer to an MS-AFLP locus to indicate a particular sized band resolved in the selective PCR. We performed selective PCR with two primer combinations.

We performed MS-AFLP following the protocol used by Richards *et al.* [9], we digested approximately 250 ng of genomic DNA at 37°C for 3 h in paired reactions; one with EcoRI and MspI, the other with EcoRI and HpaII. We immediately followed the restriction digest with adaptor ligation with EcoRI and MspI/HpaII adaptors at 16–20 h at 16°C (electronic supplementary material, table S1—all primer and adapter sequences). After adaptor ligation, we conducted pre-selective PCR with EcoRI + 1, MspI/HpaII + 0 pre-selective primers (electronic supplementary material, table S1) at the following PCR conditions: 75°C for 2 min; 20 cycles of 94°C for 30 s, 56°C for 30 s, 75°C for 2 min, final extension at 60°C for 30 min and 4°C hold. Following pre-selective PCR, we conducted selective PCR by multiplexing 6-FAM fluorescently labelled EcoRI + AGC primers with HEX fluorescently labelled EcoRI + ACG primers and unlabelled primers HpaII/MspI + TCAT (electronic supplementary material, table S1) at the following PCR conditions 94°C for 2 min, 8 cycles of 94°C 30 s, 65°C 30 s 72°C 2 min (dropping the annealing temperature 1° each cycle), 31 cycles of 94°C 30 s, 56°C 30 s 72°C 2 min, final extension of 60°C 5 min and a 4°C hold. We sent the selective PCR products to the Georgia Genomics Facility (University of Georgia) for fragment analysis on an ABI 3130XL. We used Peakscanner v. 1.0 (Applied Biosystems) to analyse resultant gel files and define fragment sizes and Rawgeno [31] to define particular bands. We duplicated the entire protocol for at least two individuals from each location to identify bands that consistently occurred and we eliminated bands that inconsistently amplified or occurred at highly variable intensities. We pooled data into two categories: methylated (types II and III) or not methylated (types I and IV).

### 2.4. Genetic population structure analysis

To assess genetic population structure, the R package *adegenet* [32,33] was used for a correspondence analysis (CoA) of microsatellite data for all sites; this multi-variate approach uses a summary of sample site allele frequencies to create a distance matrix that is used to generate principal component (PC) values for each locality, similar to a principal coordinate analysis (PCoA) of individuals. Our CoA used five PCs because this was significantly less than the number of sample localities (PCs must be less than *n*), and these five were enough to explain almost all the variance in the data. Visual clusters of sampling localities were identified. A discriminant analysis of principal components (DAPC) was then used to test if these population clusters fitted with the genotypes of individuals. The population cluster labels that were identified were used in a DAPC in *adegenet* [34,35]. This method used the individual data to calculate the percentage of individuals that were correctly assigned to their population clusters identified from the CoA using their genotypes. We choose to use the CoA of sample site (that uses allele frequencies, also accounting for the presence and absence of alleles) to define the main genetic clusters, because we predict founder effects will have had the clearest effect on allele frequency and allelic diversity between localities.

### 2.5. Epigenetic and genetic differentiation

We estimated epigenetic and genetic differentiation as Φ_ST_ among different sites, and among different introduction events using the AMOVA framework of GENALEX-6. GENALEX6 was also used to produce pairwise Φ_ST_ matrices [36], and we performed calculations over all loci and pairwise between sites in both epigenetic and genetic analyses. For all AMOVA analyses, statistical significance was estimated after 9999 permutations. We used a sequential Bonferroni correction of *α* = 0.05 for multiple tests.

We compared pairwise Φ_ST_ using a Mantel's test in R [33] using the function ‘mantel.randtest’ in the package *ade4* [37]. Finally, we compared epigenetic differentiation to geographical distance among sites using a Mantel's test.

### 2.6. Epigenetic and genetic diversity

We calculated epigenetic haplotype diversity (epi-h) and the proportion of polymorphic loci (%Poly) to characterize epigenetic diversity for each site and for each cluster, using GENALEX6 [36]. We conducted all analyses using a binary haplotype-binding pattern for 41 verified, consistent banding sites between 75 and 450 bp. %Poly represents how much ‘raw’ epigenetic variation is present in one site/cluster (i.e. the percentage of loci that are polymorphic out of the total 41 loci in our study), whereas epi-h represents haplotype diversity between the epigenetic profiles of all individuals within a site/cluster (i.e. the probability that two individuals in a site have different epigenetic profiles).

For microsatellite data, the mean number of alleles (Na) (used for ‘among cluster’ analyses), mean allelic richness (Ar) (used for ‘among site’ analyses; Ar is the equivalent of Na, but adjusted for site sample sizes) and mean expected heterozygosity (He) were calculated for each site using Fstat v. 2.9 [38].

We compared the pattern of change between MS-AFLP-based estimates of epigenetic diversity (epi-h and %Poly) with genetic characteristics of diversity (He and Ar) based on microsatellite loci genotyped in individuals from the same sample site (table 1). We initially compared estimates of epi-h/%Poly and He/Ar between all sites in our study; however, because our analyses of differentiation identified three separate clusters contingent on introduction history, we then compared levels of diversity separately among sites within the three clusters. All linear regressions were conducted in R.

### 2.7. Sample sizes

We attempted to assay the level of DNA methylation in 24 individuals of 16 populations (except Coober Pedy in which only 20 individuals were sampled). From these 380 individuals attempted, we were only successful in genotyping 180 individuals in total (47% of the individuals targeted). This rather high failure rate is owing to the difficulty of the AFLP technique used, and the sensitivity of the restriction digest step to DNA concentration. This failure rate was distributed across all sample populations (sample sizes given in table 1), except Armidale for which we were only successful in scoring four individuals. As a result, we excluded this population from all subsequent epigenetic analyses.

## 3. Results

### 3.1. Epigenetic and genetic differentiation

**Figure 2.**
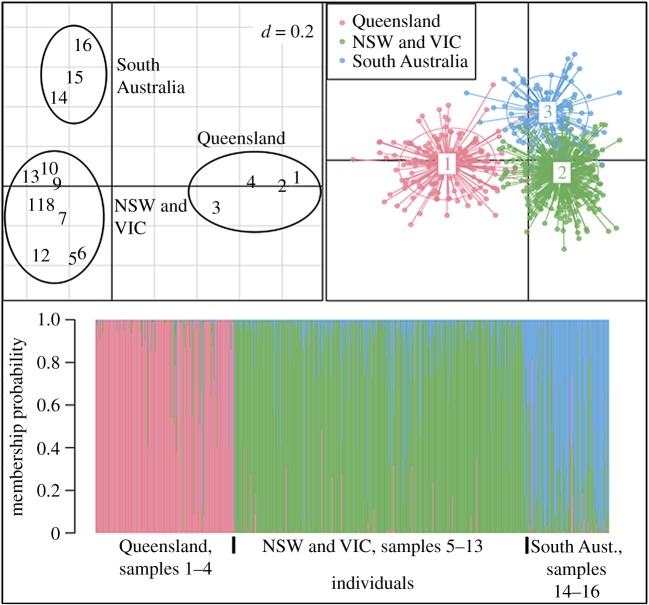
(*a*) The scatter plot for the CoA of the 16 sample localities with the three clusters that were identified. (*b*) The scatter plot from the DAPC which used the three population labels with the individual genotypes (*n* = 623 individuals). (*c*) The membership probabilities for the DAPC in (*b*). The sample labels 1–16 correspond to the sampling localities: Tolga, Townsville, Charleville, Pittsworth, Armidale (removed from epigenetic analyses owing to low sample sizes), Dubbo, Cobar, Wentworth, Burrumbuttock, Melbourne, Geelong, Bridport, Mt Gambier, Broken Hill, Adelaide and Coober Pedy, respectively (table 1).

From the microsatellite data, three population clusters were identified across our sample sites (table 1 and figure 2). These three clusters are consistent with three independent introductions to Melbourne (‘NSW and VIC’ cluster), Brisbane (‘Queensland’ cluster) and Adelaide (‘South Australian’ cluster). The percentage of individuals that were correctly assigned to each cluster in the DAPC analysis was 95.7%, 95.3% and 76.7%, respectively (figure 2*c*).

An AMOVA found that a larger portion of genetic differentiation was partitioned between the introduction events compared to between sample sites (among introduction events Φ_ST_ = 0.041, *p* < 0.001 and among sample sites Φ_ST_ = 0.025, *p* < 0.001, respectively). An AMOVA using the epigenetic data found the opposite pattern with a larger portion of epigenetic differentiation being partitioned between sample sites (Φ_ST_* *= 0.139, *p* < 0.01) compared to between introduction events (Φ_ST_* *= 0.023, *p* = 0.001).

### 3.2. Pairwise comparisons of epigenetic (Φ_ST_) and genetic (Φ_ST_) differentiation

**Figure 3.**
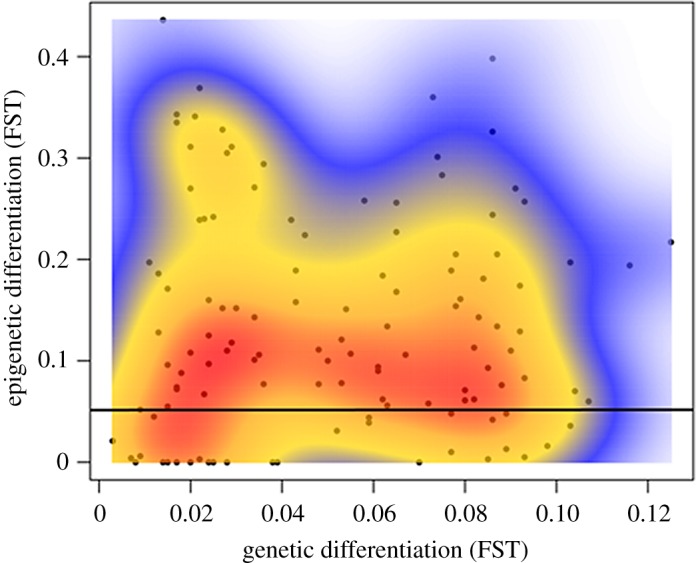
Mantel's test comparing genetic and epigenetic pairwise estimates of Φ_ST,_ across all sample sites; there is no relationship (*R^2^* = 0.124, *n* = 15, *p *= 0.159).

Pairwise comparisons of Φ_ST_ for epigenetic data were significant in 89 out of 120 cases (table 2), whereas pairwise comparisons of Φ_ST_ for genetic data were significant in 117 out of 120 cases (table 2). We found no overall relationship between pairwise comparisons of epigenetic and genetic data (Mantel's test *r* = 0.124, *n* = 15, *p *= 0.159; figure 3). The overall pattern of epigenetic differentiation was also not related to geographical distance between sites (Mantel's test *r* = −0.082, *n* = 15, *p* = 0.733).

**Table 2.** Pairwise comparisons of epigenetic (below the diagonal) and genetic (above the diagonal) variation between all sample sites (120 pairwise comparisons and 15 sites). (Bold values indicate significant Φ_ST_ values (*α* < 0.05), such that two sites are differentiated by epigenetic and genetic variation. Grey boxes represent pairwise comparisons between sample sites within genetic populations linked to the same introduction event: lightest grey indicates pairwise comparisons between sample sites from the Queensland cluster; medium grey indicates pairwise comparisons between sample sites from the NSW and VIC cluster; and dark grey indicates pairwise comparisons between sample sites from the SA cluster.)

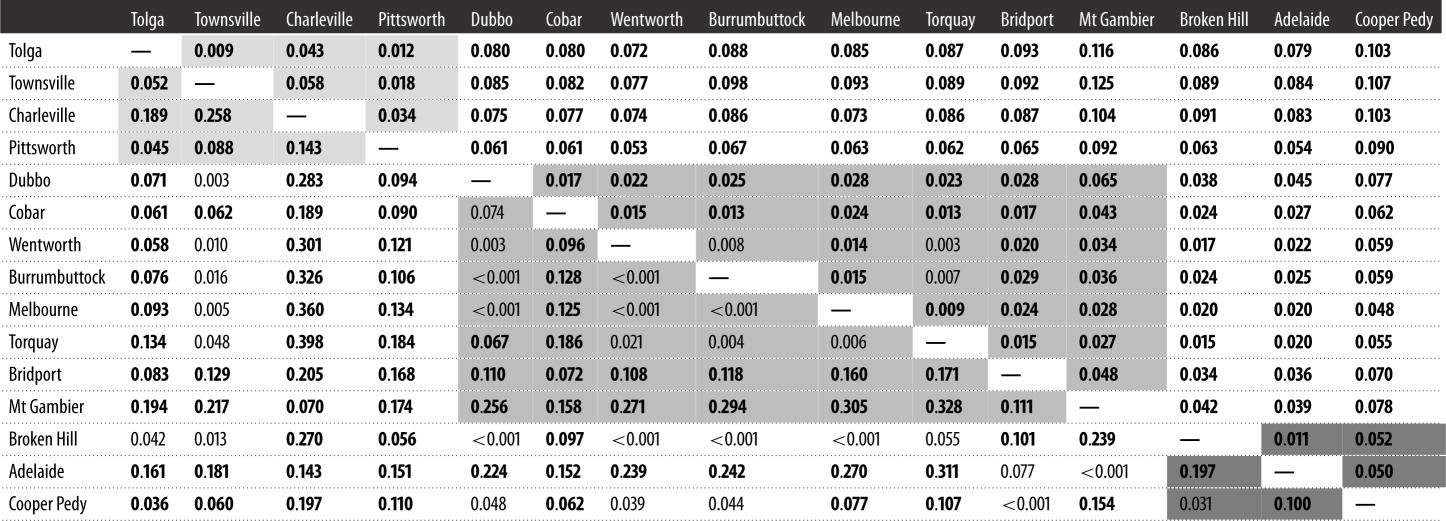

### 3.3. Relationship between epigenetic and genetic diversity

Among the 15 sites surveyed, average epi-h measures for each site ranged from 0.16 to 0.44 and average %Poly for each site ranged from 46.34 to 97.56 (table 1). The two measures of epigenetic diversity (%Poly and epi-h) were strongly related to one another (estimate = 0.005, *t*_13_ = 6.04, *p* < 0.001, *R^2^* = 0.74). Measures of genetic diversity (He and Ar) were also strongly related to each other (estimate = 31.53, *t*_13_ = 4.39, *p* < 0.001, *R^2^* = 0.60).

Although we detected a negative correlation between %Poly and He/Ar across all sites (*n* = 15) (electronic supplementary material, figure S1), it is important to note that these 15 sites are not independent and the populations represent three distinct clusters. When we focused on the relationships between epigenetic and genetic diversity within the three introduction clusters, no correlations were detected. Between sites in the NSW/VIC introduction (*n* = 9), there was no significant correlation between epi-h and He or Ar (Spearman's correlation: *r* = −0.610, *p* = 0.081, *r* = −0.475, *p *= 0.197, respectively), or %Poly and He or Ar (*r* = −0.617, *p* = 0.07, *r* = −0.317, *p* = 0.406); between sites in the South Australian introduction (*n* = 3), there was no significant correlation between epi-h and He or Ar (*r* = −0.500, *p* = 0.667, *r* = −0.500, *p* = 0.677), or % Poly and He or Ar (*r* = −0.500, *p* = 0.667, *r* = −0.500, *p* = 0.677); and no significant correlation was detected between epi-h and He or Ar in sites within the Queensland introduction (*n* = 4) (*r* = −0.400, *p* = 0.60, *r* = −0.60 *p* = 0.40), or % Poly and He and Ar (*r* = −0632, *p* = 0.368, *r* = 0.316, *p* = 0.684, respectively). However, all but the last of these correlations is negative indicating a consistent pattern that could be non-significant in each case owing to the small sample sizes.

The non-independence of the multiple sites sampled within the three introduction clusters suggests that the most appropriate way to analyse the results is to compare the level of genetic diversity and epigenetic diversity across the three clusters (i.e. reducing the overall sample size to just three points (the mean of each cluster)), with each having a reasonable sample of individuals (between 29 and 90 individuals). Between the three clusters, there was a significant negative correlation between Na and epi-h (Spearman's correlation: *r *= 0.995, *p* = 0.015), and a negative (but non-significant) trend between: He and epi-h (*r* = −0.763, *p* = 0.462), Na and %Poly (*r *= 0.577, *p* = 0.608) and He and %Poly (*r* = 0.974, *p* = 0.146); however, none of these results were significant after the Bonferroni correction for the multiple tests conducted.

## 4. Discussion

Epigenetic mechanisms have been hypothesized to contribute to the diversity and evolutionary potential of introduced populations, which must adjust to novel habitats with relatively low levels of genetic variation [10]. To gain insight into how epigenetic mechanisms are associated with different introduction events, here, we characterize patterns of DNA methylation in multiple, highly successful introductions of house sparrows into Australia *ca.* 160 years ago.

Using genetic data from our sample sites, we find three major population clusters across the Australian house sparrow population, that are consistent with the historical descriptions of three independent introductions [25,26]. These genetic results confirm how our sample sites should be grouped when assessing the epigenetic data, and indeed, we find significant epigenetic differentiation between these clusters. Interestingly however, a hierarchical AMOVA found that epigenetic differentiation was stronger among sample sites than among invasion clusters, whereas the opposite pattern was found in the genetic data (where more differentiation was detected between invasion clusters). These findings suggest that patterns of epigenetic variation are more strongly influenced by local environmental stimuli or sequential founder events than the initial diversity in the introduction population. Additional samples from each of our study sites may provide greater confidence in the population differences in the epigenetic marks, and would allow for an investigation of potential environmental or ecological drivers of the observed patterns of variation.

In addition to the patterns of epigenetic differentiation identified within the established Australian house sparrow population, epigenetic differentiation between populations has also been detected among different subspecies of the house sparrow in its established native range [39]. Contrarily, no epigenetic differentiation has been detected throughout the Kenyan population of house sparrows, during its initial stages of invasion [10]. During the initial stages of invasion, rates of dispersal and environmental change may operate too rapidly for stable epigenetic marks to differentiate to location, or selection may exist for the maintenance of liable epigenetic marks (to facilitate diversifying bet-hedging strategies [22]). Sample sizes for the assay of epigenetic variation were much lower in each of the populations studied in the initial stages of the Kenyan invasion [10]; thus, further work is necessary to clarify the influence of population age on epigenetic differentiation.

In our study, while overall patterns of epigenetic and genetic differentiation were similar, Mantel's tests indicated that no correlation existed between pairwise site comparisons of epigenetic and genetic differentiation. These results suggest that at least some of the observed differentiation in DNA methylation has arisen independently or at least partly independently from genetic differentiation. Significant correlations between epigenetic and genetic differentiation have been reported in a range of plants[40], and animals [41,42]. However in line with our results, an increasing number of studies also find no correlations between epigenetic and genetic differentiation, [9,43–45] which may suggest a role for epigenetic mechanisms to act as alternative sources of variation.

We did detect significant variation in the level of epigenetic diversity at each sample site throughout Australia (epi-h range = 0.16–0.44), and this is consistent with reports of high levels of epigenetic diversity across other invasive house sparrow populations (e.g. in Kenya: epi-h range = 0.28–0.44 [10]; and North America: epi-h range = 0.35–0.36 [1]). Previous research has also identified a negative relationship between epigenetic and genetic diversity in introduced house sparrows [10]. In the present study, a negative trend between epigenetic and genetic diversity was found between the invasion clusters; however, these inter-invasion cluster analyses had low statistical power and none were significant. Before partitioning the Australian house sparrow population into its three distinct clusters, we also found the same negative trend between epigenetic and genetic diversity between all 15 sites (this relationship was significant for the two %Poly models and nearing significance for the epi-h models (electronic supplementary material, figure S1)). These correlations, however, probably represent an artefact of differences between the three clusters (probably arising from founder effects); thus, our results emphasize the need to incorporate invasion history into population-wide analyses of epigenetic patterns, to avoid pseudo-replication in samples of populations that have derived from the same introduction event. Further work is needed to understand the dynamics between epigenetic and genetic diversity; however, elevated epigenetic diversity is likely to only compensate for reduced levels of genetic diversity when genetic diversity is lowest immediately after a bottleneck, and invaders are facing the most novel environments (i.e. before they have established). Consequently, while our results are only suggestive of a relationship between epigenetic and genetic diversity in Australian house sparrows, such a relationship may have been stronger in earlier stages of the introduction, and is now obscured by recovered genetic diversity and generally more established population dynamics.

The potential for epigenetic patterns to facilitate invasive success requires future inquiry, and our data highlight the importance of identifying epigenetic patterns in multiple and independent invasive expansions before they can be implicated in facilitating invasive expansions. We conclude that a negative, compensatory relationship between epigenetic and genetic diversity does not currently exist within the Australian house sparrow range. However, future work should assess the extent to which this relationship may exist in populations at more initial stages of an invasion when genetic diversity is likely to be lowest, and the need to respond to novel environments is likely to be highest. It is also possible that low sample sizes have hindered our ability to detect a significant correlation between genetic and epigenetic variation. Future work should repeat our analyses by increasing the number of individuals that are screened for variation in DNA methylation.

Our detection of significant patterns of epigenetic differentiation over such broad geographical areas across Australia may indicate that stable transmission of epigenetic marks is possible, or that epigenetic marks respond reliably to consistently different environments or founding events. An experimental approach is required to determine the extent of stable transmission of DNA methylation states in house sparrows (and thus the extent to which epigenetic mechanisms may contribute to evolution via natural selection), but stable transmission has been demonstrated in other taxa (i.e. [19,46]). Our results provide a foundation for future work to examine the phenotypic and evolutionary relevance of epigenetic variation, potentially through combining next-generation sequencing techniques (that elucidate genome-wide patterns of DNA methylation) with RNAseq estimates of gene expression, and ecologically relevant phenotypes [14,15].

## Supplementary Material

Supplementary-Figure 1

## Supplementary Material

Supplementary Table 1.

## Data Availability

We deposited all genetic and epigenetic binary haplotype data on Dryad http://dx.doi.org/10.5061/dryad.44dm3 [46].
