# Supplementary material for: Epigenetic and genetic variation among three separate introductions of the house sparrow (Passer domesticus) into Australia
Source: R Soc Open Sci. 2018 Apr 11;5(4):172185. doi: 10.1098/rsos.172185 (PMC5936936; doi:10.1098/rsos.172185)
Supplement: Supplementary-Figure 1 [file rsos172185supp1.pdf]

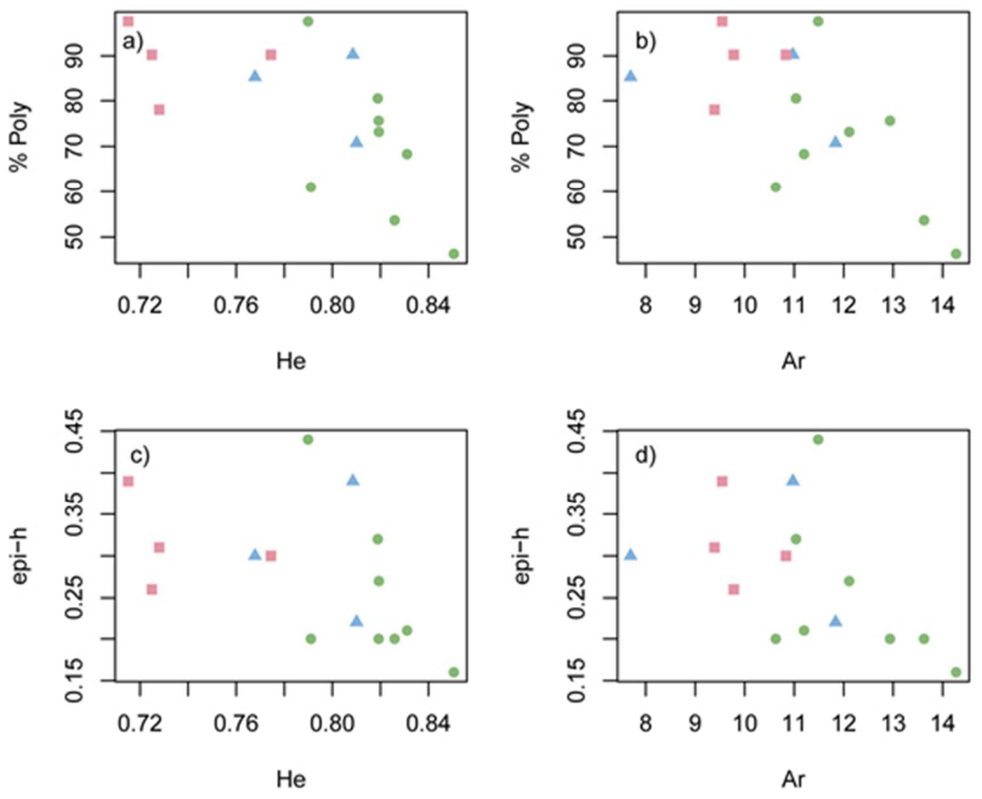

Supplementary-Figure 1. Across all sites (n=15) we detected a significant negative relationship between %Poly and He /Ar. (%Poly and He = -241.16, t13 = -3.10, P = 0.006, R2 = 0.43; %Poly and for Ar = -5.88, t13 = -3.07, P = 0.007, R2 = 0.42), epi-h also had a trend showing a negative relationship (that approached significance) with He and Ar (epi-h and He = -0.96, t13 = -1.99, P = 0.069, R2 = 0.23; epi-h and Ar = -0.02, t13 = -2.06, P = 0.06, R2 = 0.25. Epigenetic diversity (% Poly =percentage of polymorphic MS-AFLP loci, epi-h=epi-haplotype diversity), genetic diversity (He= expected heterozygosity and Ar= allelic richness). Pink squares: Queensland introduction; Green circles: Melbourne introduction; Blue triangles: South Australian introduction.

151x127mm (96 x 96 DPI)
