# Supplementary material for: Epigenetic and genetic variation among three separate introductions of the house sparrow (Passer domesticus) into Australia
Source: R Soc Open Sci. 2018 Apr 11;5(4):172185. doi: 10.1098/rsos.172185 (PMC5936936; doi:10.1098/rsos.172185)
Supplement: Supplementary Table 1. [file rsos172185supp2.pdf]

Primer/Adapter Name	Sequence (5’ to 3’)
EcoRI Adapter Forward	CTCGTATACTGCGTACC
EcoRI Adapter Reverse	AATTGGTACGCAGTA
MspI/HpaII Adaptor Forward	GATCATGAGTCCTGCT
MspI/HpaII Adaptor Reverse	CGAGCAGGACTCATGA
EcoRI Pre-selective Primer	TACTGCGTACCAATTCA
MspI/HpaII Pre-selective Primer	ATCATGAGTCCTGCTCGG
EcoRI Selective Primer	6-FAM-TACTGCGTACCAATTCAGC
EcoRI Selective Primer	5HEX-TACTGCGTACCAATTCACG
MspI Selective Primer	ATCATGAGTCCTGCTCGGTCAT
